# Novel electrospun poly-hydroxybutyrate scaffolds as carriers for the wound healing agents alkannins and shikonins

**DOI:** 10.1093/rb/rbab011

**Published:** 2021-06-10

**Authors:** Athanasios S Arampatzis, Konstantina Giannakoula, Konstantinos N Kontogiannopoulos, Konstantinos Theodoridis, Eleni Aggelidou, Angélique Rat, Elli Kampasakali, Anne Willems, Dimitrios Christofilos, Aristeidis Kritis, Vassilios P Papageorgiou, Ioannis Tsivintzelis, Andreana N Assimopoulou

**Affiliations:** 1 Laboratory of Organic Chemistry, School of Chemical Engineering, Aristotle University of Thessaloniki (AUTh), Thessaloniki 54124, Greece; 2 Natural Products Research Centre of Excellence (NatPro-AUTh), Center of Interdisciplinary Research and Innovation of Aristotle University of Thessaloniki (CIRI-AUTh), Thessaloniki 57001, Greece; 3 Department of Physiology and Pharmacology, School of Medicine, Faculty of Health Sciences, Aristotle University of Thessaloniki (AUTh), Thessaloniki, Greece; 4 Laboratory of Microbiology, Faculty of Sciences, Ghent University, Ghent 9000, Belgium; 5 Faculty of Engineering, School of Chemical Engineering and Physics Laboratory, Aristotle University of Thessaloniki, Thessaloniki 54124, Greece; 6 Physical Chemistry Laboratory, School of Chemical Engineering, Aristotle University of Thessaloniki, Thessaloniki 54124, Greece

**Keywords:** electrospinning, polyhydroxyalkanoates, alkannin, shikonin, wound healing

## Abstract

The aim of this study was to investigate the potential of novel electrospun fiber mats loaded with alkannin and shikonin (A/S) derivatives, using as carrier a highly biocompatible, bio-derived, eco-friendly polymer such as poly[(R)-3-hydroxybutyric acid] (PHB). PHB fibers containing a mixture of A/S derivatives at different ratios were successfully fabricated via electrospinning. Αs evidenced by scanning electron microscopy, the fibers formed a bead-free mesh with average diameters from 1.25 to 1.47 μm. Spectroscopic measurements suggest that electrospinning marginally increases the amorphous content of the predominantly crystalline PHB in the fibers, while a significant drug amount lies near the fiber surface for samples of high total A/S content. All scaffolds displayed satisfactory characteristics, with the lower concentrations of A/S mixture-loaded PHB fiber mats achieving higher porosity, water uptake ratios, and entrapment efficiencies. The *in vitro* dissolution studies revealed that all samples released more than 70% of the encapsulated drug after 72 h. All PHB scaffolds tested by cell viability assay were proven non-toxic for Hs27 fibroblasts, with the 0.15 wt.% sample favoring cell attachment, spreading onto the scaffold surface, as well as cell proliferation. Finally, the antimicrobial activity of PHB meshes loaded with A/S mixture was documented for *Staphylococcus epidermidis* and *S. aureus*.

## Introduction

Human skin could be considered as body’s first line of defense against various environmental factors performing several functions, such as protection from pathogenic microorganisms and thermoregulation. Being the outermost layer of the body, skin is in continuous contact with the outer environment, rendering susceptible to injuries. Hence, when the skin is harmed, it should be secured by the appropriate wound dressing (depending on the severity of the wound), aiming to enhance fast regeneration and avoid unwanted complications, such as infections etc. [1, 2].

During the past decade, significant advances and progress have been made concerning wound care applications, moving on from conventional treatments (ointments and gauze bandages) to the use of advanced multifunctional wound dressings (e.g. biodegradable patches or porous matrices). When such dressings are applied on the wound, they offer protection from wound dehydration and pathogenic invasion, thus facilitating faster wound healing [3]. They only provide a passive protection, unless they are loaded with active pharmaceutical ingredients (APIs), which render them active wound dressings (AWDs) that can protect the wound bed, inhibit pathogenic proliferation, and accelerate the process of healing [4, 5]. Furthermore, AWDs should preferably offer more sophisticated properties, such as biocompatibility and nontoxicity, absorption of accumulated wound exudates, thermal and mechanical resistance, sterile and nonallergic environment, nonsensitizing, and ability to maintain a prolonged drug release to the site of injury [6].

Electrospinning is a simple and effective technique to fabricate drug-loaded micro- or nano-scale polymeric fiber-based non-woven mats, using a wide range of natural and synthetic polymers. Morphologically, the electrospun fibers resemble the extracellular matrix (ECM) of the native tissue, exhibiting high surface to volume ratio, variable pore size, high porosity, and oxygen permeability, helping in this way the proper proliferation of the cells [1, 7, 8].

**Figure 1.**
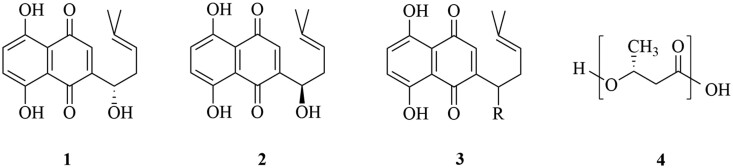
Structures of alkannin (1), shikonin (2) and their derivatives (3), as well as poly[(*R*)-3-hydroxybutyric acid] (PHB) (4).

Various synthetic and natural biodegradable polymers can be used for the fabrication of electrospun nanofibers. However, biopolymers attracted particular attention for biomedical applications, due to their low toxicity, immunogenicity and improved biocompatibility compared to synthetic polymers. Biodegradable polymers are preferable as they offer the ability to control the release rate of the drug, either by diffusion through the polymer matrix (or the pores within the matrix), or/and by degradation of the polymer chains and erosion of the matrix [9–11]. Polyhydroxybutyrate (PHB, Fig. 1), the main representative of polyoxyalkanoates (PHA), is a bio-derived, ‘green’ and low-cost hydrophobic biocompatible polymer with a high degree of crystallinity. It is obtained from natural sources, namely from certain bacteria, that produce it through fermentation of sugars and fatty acids at times of excess carbon source. PHAs are accumulated as inclusions in the cells to serve as a carbon and energy reserve in circumstances of nutrient deficiency. Due to its natural origin, excellent biocompatibility, biodegradability and non-toxicity, PHB is an appealing agent for various biomedical applications, including drug delivery systems (DDSs) and tissue engineering, and consequently, attracted commercial attention. It has been evaluated for controlled drug release systems, surgical sutures, wound dressings, orthopaedic devices, tissue engineering and skin substitute materials, and used for the fabrication of electrospun drug-loaded fiber mats [11–14].

Some of the desirable features for wound dressings are protection of wounds/ulcers against infection, tissue regeneration and epithelization. Having that in mind, in this study we relied on alkannin and shikonin (A/S) derivatives (Fig. 1) as APIs, aiming to benefit from their strong wound healing, regenerative and antimicrobial activity [15].

Extensive scientific investigations in the Organic Chemistry Laboratory of the School of Chemical Engineering at AUTh over the past 40 years have substantiated the biological activity of Alkannins and Shikonins, as well as their antioxidant, anti-inflammatory, tissue regenerative and most prominently, antitumor action. Clinical studies have revealed that they belong to a very small group of therapeutics that modulate both the inflammatory and proliferative phases of wound healing, while delivering an antimicrobial and analgesic effect in a broad range of ulcers (i.e. indolent and chronic ulcers, leprotic ulcers, burns and anal fissures) [15–18]. It is important to mention that wound healing pharmaceutical ointments with A/S and their derivatives as APIs, invented by Prof. Papageorgiou of our group, have been authorized by the National Organization of Medicines in Greece and are commercially available under the trademarks HELIXDERM^®^ and Histoplastin Red^®^. The mixture of A/S derivatives, mainly esters, isolated from roots of the pharmaceutical plant *Alkanna tinctoria*, has been proven more active than each pure compounds individually, regarding the wound healing and antimicrobial activity [15–17]. Furthermore, it can be isolated more easily using a few-steps procedure and at a lower cost compared with pure compounds, diminishing the overall cost of the final biomaterial.

To the best of our knowledge, this work is the first study on the fabrication of PHB electrospun fiber mats loaded with a mixture of A/S derivatives (A/S mixture) as API for potential wound dressing applications. Based on our previous knowledge and related expertise [19], we used different initial concentrations of drug (0.15, 0.5, 3.2, 6.9, and 13.7 wt.% based on the polymer weight) to evaluate the capacity of the PHB fibrous membranes to incorporate the A/S mixture. The morphology of the fabricated mats was investigated using scanning electron microscopy (SEM) and their physicochemical characteristics by employing differential scanning calorimetry (DSC) and thermogravimetric analysis (TGA). Raman, IR and luminescence spectroscopies provided further information on the crystallinity and drug loading, while their pharmacokinetic characteristics were studied in terms of entrapment efficiency and *in vitro* drug release. Furthermore, after performing preliminary experiments, the fiber mats with 0.15, 0.5 and 6.9% A/S mixture were assessed for their biocompatibility with Hs27 fibroblasts, in terms of cell morphology, viability and attachment, whereas all samples were tested for their antimicrobial activity against *Staphylococcus epidermidis* and *Staphylococcus aureus*, in order to evaluate the possibility of the A/S mixture-loaded PHB fibrous mats to act as potential wound dressing materials.

This study is a continuation of the authors’ research on exploiting the biological properties of A/S and other naphthoquinones via the preparation of sophisticated DDSs and specifically electrospun fiber mats, contributing to the field of tissue and skin engineering through the preparation of biodegradable and biocompatible wound healing dressings promising for the repair and regeneration of the skin.

## Materials and methods

### Materials

Poly[(*R*)-3-hydroxybutyric acid] and sodium lauryl sulfate (SLS) were purchased from Sigma-Aldrich (St. Louis, MO). The mixture of A/S pigments (A/S mixture) was isolated from *A. tinctoria* roots obtained from Soft-N-Supple (Punjab, Pakistan), according to the isolation and purification workflow proposed by Prof. Papageorgiou [20]. The A/S mixture was subjected to HPLC-DAD (Agilent Technologies, Waldbronn, Germany) analysis and the corresponding A/S derivatives were determined. Human fibroblasts Hs27 (ATCC^®^ CRL-1634™) were used in cell culture experiments and purchased from the American Type Culture Collection (ATCC, Manassas, VA). Dulbecco’s Modified Eagle’s Medium (DMEM), fetal bovine serum (FBS), penicillin/streptomycin, trypsin-EDTA, glutaraldehyde and osmium tetroxide were purchased from Sigma-Aldrich (St. Louis, MO). 3-(4,5-Dimethylthiazol-2-yl)-2,5-diphenyltetrazolium bromide (MTT) was obtained from Sigma-Aldrich (St. Louis, MO). Viability/Cytotoxicity Assay Kit for Animal Live & Dead Cells was purchased from Biotium (CA). *Gram*-positive *S. epidermidis* (LMG 10474) and *S.* aureus (LMG 8224) were obtained from the Belgian Co-ordinated Collections of Micro-organisms (BCCM, Ghent, Belgium). Muller-Hinton (MH) agar and nutrient agar (NA) were purchased from Merck (Darmstadt, Germany). Dimethyl sulfoxide (DMSO) was purchased from AppliChem (Darmstadt, Germany). Phosphate-buffered saline (PBS) was purchased from Gibco^®^ (Thermo Fisher Scientific, Waltham, MA). All reagents used in the experiments were of analytical grade. Chloroform and ethanol were purchased from Fisher Scientific (Pittsburgh). *N, N*-dimethylformamide (DMF) and diethyl ether were obtained from Honeywell (Charlotte, NC).

### Electrospinning experiments

Polymer solutions were prepared at a fixed concentration of 6.0% w/w PHB in 9:1 v/v chloroform/DMF (concentrations that were selected according to preliminary electrospinning experiments). Before the addition of the A/S mixture, the polymer-solvent mixtures were stirred for 6 h and heated to approximately 70°C for 20 min to obtain a homogenous solution. Subsequently, the A/S mixture was added into the polymer solutions at various concentrations. Compositions of different experiments are shown in Table 1. The electrospinning setup is a homemade system. The apparatus consists of a syringe pump (HARVARD APPARATUS, model 2274) for the injection of the polymer solution, a rotary drum covered with aluminum foil as a grounded substrate for the collection of the fibers and a high voltage power supply (Spellman High Voltage DC SUPPLY, model RHR30P30). All experiments were performed using a glass syringe of 5 mL internal volume and 1 mm (18 G) needle diameter. The flow rate of the polymer solution was controlled by the syringe pump and was set to 0.7 mL/h. The needle was connected to the high-voltage supply and the ground electrode was connected on the conductive aluminum surface. The distance between the needle and the collector was 9 cm and the applied voltage was 15 kV (conditions that were selected after performing preliminary tests). Experiments were performed at ambient temperature (22–25°C). All the obtained electrospun samples were vacuum-dried (320 Pa, Shanghai Laboratory Instrument Work Co. Ltd., Shanghai, China) for 24 h at 50°C in order to remove the residual organic solvents and moisture [19].

**Table 1.** Composition of the electrospun fiber mats.

Sample	PHB solution (% w/w)	A/S mixture (wt.%)^a^
Neat	6.0	—
1	6.0	0.15
2	6.0	0.5
3	6.0	3.2
4	6.0	6.9
5	6.0	13.7

^a^ Based on the polymer weight.

### Characterization of the electrospun fibers

#### Morphological analysis by SEM

Samples of the fibrous structures were examined by SEM (JEOL, mod. JSM-6390LV, Japan) located at the Electron Microscopy Laboratory, School of Physics, Aristotle University of Thessaloniki. All surfaces were coated with graphite to avoid charging under the electron beam. For each fibrous membrane, two independent samples were examined under the microscope at three representative areas and three magnifications were recorded for each area (1000×, 2000× and 4000×). Appropriate software (ImageJ software, National Institutes of Health) was used to analyze the images and evaluate the fiber diameter distribution and the mean diameter for each sample (400–500 diameter measurements in average).

#### Porosity assessment

The porosity of the scaffolds was determined according to the liquid displacement method [21]. Briefly, each sample (1 × 1 cm) was weighed and immersed in a graduated cylinder filled with 7 ml ethanol (V_1_). The resultant volume was recorded as V_2_. After 10 min, the sample was removed and the remaining volume of ethanol was recorded as V_3_. The porosity was calculated by the following equation:

(1) $$\mathrm{Porosity}\;(\%)=\left(\frac{V_{1}\;–\;V_{3}}{V_{2}\;–\;V_{3}}\right)\;\times \;100$$

#### Water uptake ratio evaluation

The water uptake of PHB and A/S mixture-loaded PHB scaffolds was determined by incubating the samples in distilled water at room temperature for 24 h. Briefly, three independent fiber mats were cut into 1 × 1 cm pieces and their dry weight was weighed (*W*_0_). Next, the samples were immersed in 2 ml distilled water and after 24 h their wet weight was measured again (*W*_1_). The water uptake capacity was calculated as follows:

(2) $$\mathrm{Water}\;\mathrm{uptake}\;(\%)=\left(\frac{W_{1}\;–\;W_{0}}{W_{0}}\right)\;\times \;100$$

#### Entrapment efficiency and drug loading

Entrapment efficiency, representing the drug incorporated in the composite polymer matrix both inside the fibers and conjoined on their surface compared to the initially used drug, was quantified by UV/Vis spectrophotometry (UV–1900 spectrophotometer, Hitachi, Japan) at 516 nm. A pre-weighed amount of fiber mat (3–5 mg) was diluted in 5 ml chloroform (under vortexing) in order to dissolve the fiber structure, releasing the drug (A/S mixture) into the organic solvent. The suspension was filtered and its absorbance was measured spectrophotometrically (control: chloroform, total A/S content). A calibration curve of various concentrations (*n* = 9) of the A/S mixture in chloroform *vs* absorbance values was constructed to estimate drug content:

(3) $$\mathrm{Drug}\;\mathrm{concentration}\;(\mathrm{mg}/\mathrm{mL})\;=\;0.0411\;\times \;\mathrm{absorbance}\;-\;0.0002;\;(R^{2}=0.996)\;$$

The entrapment efficiency and drug loading of the fibers were calculated using the following equations:

(4) $$\mathrm{Entrapment}\;\mathrm{Efficiency}\;\left(\mathrm{EE}\right)\;\left(\%\right)=\left(\frac{\mathrm{amount}\;\mathrm{of}\;\mathrm{drug}\;\mathrm{incorporated}\;\mathrm{into}\;\mathrm{fibers}}{\mathrm{initially}\;\mathrm{added}\;\mathrm{amount}\;\mathrm{of}\;\mathrm{drug}}\right)\;\times \;100$$

(5) $$\mathrm{Drug}\;\mathrm{loading}\;\left(\%\right)=\left(\frac{\mathrm{amount}\;\mathrm{of}\;\mathrm{drug}\;\mathrm{incorporated}\;\mathrm{into}\;\mathrm{fibers}}{\mathrm{weight}\;\mathrm{of}\;\mathrm{fibers}}\right)\;\times \;100$$

Furthermore, in order to determine the uniformity of the drug content throughout each sample, the entrapment efficiency and loading from random parts of the polymeric construct was calculated as described above. Three independent meshes were analyzed each time in triplicate (*n* = 9 for each sample) and the average values (±SD) were calculated.

#### Spectroscopic techniques

Raman and luminescence measurements were conducted on the as-prepared samples in the backscattering geometry using a LabRAM HR (Horiba, Japan) single stage, micro-Raman spectrometer, equipped with a Peltier-cooled CCD detector. The instrument incorporates an u-Eye (IDS, Germany) optical camera that in combination with its microscope allows sample visualization and selection of the areas of interest for probing. The optical images presented in this work were captured with this device.

For the excitation of both Raman and luminescence spectra, a Fandango (Cobolt, Sweden) diode-pumped solid-state laser at 515 nm was employed. The spectral resolution of the system was ∼3.5 cm^−1^. For the Raman spectra, a standard 100×, N.A. 0.9 objective was used, focusing the laser radiation on a spot with diameter of ∼1.0 μm. The presented spectra from PHB powder and the neat fiber mats were acquired using a power of ∼1 mW. In order to collect the signal from the photosensitive drug molecules inside the drug-loaded fibers, the laser spot with a power of only ∼3 μW was rapidly scanned along the probed fiber using the DuoScan^®^ (Horiba, Japan) system, averaging signal from a fiber segment of ∼10 μm in length. Using the same beam-scanning system, luminescence spectra were collected from an area of 20 × 20 μm^2^ on the samples, using a super long working distance 50×, N.A. 0.45 objective and a laser power of ∼0.3 μW.

IR spectra of all the samples were acquired in attenuated total reflection (ATR) mode by means of a IR-Prestige-21 FT-IR spectrometer (Shimadzu, Japan) located at the Department of Pharmaceutical Technology, School of Pharmacy, Aristotle University of Thessaloniki. FT-IR spectrometer was coupled with a horizontal Golden Gate MKII single reflection ATR system (Specac, UK), equipped with ZnSe lenses. Spectra were obtained in the region of 750–1900 cm^−1^. Sixty-four (64) scans over the selected wavenumber range at a resolution of 4 cm^−1^ were averaged for each sample.

#### Thermal analysis

Calorimetric measurements were carried out with a Shimadzu differential scanning calorimeter (DSC-50, Shimadzu, Japan). All measurements were performed using a heating rate of 10°C min^−1^ under a constant nitrogen flow of 20 cm^3^ min^−1^. The sample weight was kept at low levels (<5 mg) in order to minimize any possible thermal lag during the scans. Thermal gravimetric analysis was performed in a Shimadzu TGA-50 thermogravimetric analyzer, using a heating rate of 10°C min^−1^ and a constant N_2_ flow rate of 20 cm^3^ min^−1^.

#### *In vitro* dissolution studies

*In vitro* dissolution studies were performed according to our previously published study [19]. In brief, a drug-loaded fiber sample (25–30 mg) was incubated in 30 ml of PBS with pH 5.7 + 1% SLS at 35°C under magnetic stirring for 72 h. Aliquots of samples (3 ml) were taken from the release medium at specific time intervals, filtered and evaluated for A/S mixture content spectrophotometrically at 516 nm (UV–1900 spectrophotometer, Hitachi, Japan), while the same volume of phosphate buffer was replaced to maintain sink conditions. Drug released from the fiber mats at different timepoints was estimated through a calibration curve of various concentrations (*n* = 9) of the A/S mixture in the release medium *vs* absorbance values. Three independent fiber mats were analyzed each time for their dissolution profile in triplicate (*n* = 9 for each sample) and the average values (±SD) were calculated.

$$\mathrm{Drug}\;\mathrm{concentration}\;(\mathrm{mg}/\mathrm{mL})\;=\;0.0392\;\times \;\mathrm{absorbance}\;+\;0.0001;\;(R^{2}=1)(6)$$

### Biocompatibility evaluation

#### Cell seeding

All cell culture experiments have been approved by the Ethics and Research Integrity Committee of the Aristotle University of Thessaloniki, Greece (#254319/2020). Human normal fibroblasts (Hs27) were cultured in 75 cm^2^ cell culture flasks in high-glucose DMEM supplemented with 10% FBS, 100 units/mL penicillin and 100 μg/mL streptomycin and incubated at 37°C in 5% CO_2_ atmosphere (mod. MCO-19M, Panasonic, Japan). The medium was changed 2–3 times per week. Prior to cell seeding, electrospun scaffolds were punched into 8 mm diameter circular discs with a biopsy punch (GIMA, Italy), UV-sterilized from both sides under laminar flow hood (Class II A2 Biosafety Cabinet, mod. BSC-1300IIA2-X, Biobase, China) for 1 h in total, placed in 24-well plates and immersed in complete culture medium overnight. Hs27 cells (passages 5–8) were harvested using 0.25% trypsin-EDTA, counted with a Neubauer chamber (BRAND GmbH, Germany) and suspended in 450 μL of complete medium, at a density of 0.5 × 10^5^ cells/disc. Afterwards, the cell suspension was spread on top of the sterile fiber samples to cover completely their surfaces. Cell-seeded membranes were incubated at 37°C with 5% CO_2_ for 40 min to enable cells adhere before adding 1 ml/disc of complete medium.

#### Cell adhesion

Cell adhesion on unloaded and A/S mixture-loaded scaffolds was evaluated by confocal microscopy. To this end, the Viability/Cytotoxicity Assay Kit for Animal Live & Dead Cells was employed containing 2 μM calcein AM and 4 μM ethidium homodimer III (EthD-III). At days 3 and 7, samples were rinsed with PBS and double stained with calcein AM and EthD-III, staining living and dead cells, respectively. Subsequently, the stained samples were incubated in the dark, at room temperature for 30 min and rinsed twice with PBS, before observing them under a confocal upright microscope (D‐Eclipse 80i C1, Nikon, Japan) located in the Laboratory of Anatomy, Histology and Embryology, Faculty of Veterinary Medicine, AUTh. For the excitation of Hs27 cells, the 488 nm (for Calcein-AM) and 543 nm (for EthD-III) lasers were used, while the detection of the emitted light was performed at 520 and 617 nm, respectively. Samples were recorded as z-stack images using the EZ—C1 3.20 software. Images are in color and, in our case, the information is coded in pure green (live cells) and pure red (nucleus of dead cells) of the RGB color system [22]. Cell surface coverage for live and dead cells was calculated by finding the fraction of pixels above a threshold intensity individually for each color channel by both ImageJ and an in-house pascal code developed in ‘The professional Free Pascal RAD IDE’, Lazarus Team (1993-2020). Note that since stained areas of live and dead cells are different (the whole cell for live cells, the nucleus for dead ones), surface coverage values of live and dead cells are not directly comparable. The cell infiltration analysis was performed on z-stack images by using the 3D surface plot plugin of the software ImageJ.

#### Cell morphology

Morphology of the attached cells on fiber mats was investigated by SEM at the Electron Microscopy Laboratory, School of Physics, AUTh. At days 3 and 7, electrospun membranes were removed from the culture medium and rinsed twice with PBS. Cells were fixed on the surface of the scaffolds with 3% v/v glutaraldehyde, washed with PBS and post-fixed with osmium tetroxide. After washing with PBS, samples were dehydrated in increasing concentrations (30–100% v/v) of ethanol. Finally, the samples were dried, sputter coated with carbon and examined with SEM [23].

#### Cell viability

The effect of the A/S mixture-loaded scaffolds on Hs27 cells was determined by 3-(4,5-dimethyl-thiazoyl)-2,5-diphenyl-SH-tetrazolium bromide (MTT) assay. The cells were seeded at a density of 0.5 × 10^5^ cells/disk in 24-well plates for 3 and 7 days. After the respective incubation time periods, the seeded scaffolds were transferred to a new 24-well plate and incubated overnight at 37°C with 5% CO_2_; afterwards, cell viability was evaluated by MTT. Briefly, 400 μL of culture medium and 40 μL of MTT (at 0.5 mg/mL final concentration) were added into each well and incubated for 4 h at 37°C in 5% CO_2_ atmosphere. Afterwards, supernatants were discarded and 400 μL of SDS-HCl solution were added to each well to dissolve the insoluble formazan crystals. Aliquots of the dissolved formazan solution (100 μL) were transferred to a 96-well plate and the absorbance was measured at 570 nm using a micro-plate reader (Stat Fax—2100, Awareness Technology Inc.). Unloaded scaffolds served as controls. The results are presented relatively to the absorbance of the control samples (neat sample). This means relative mitochondrial enzyme activity in the reduction of the MTT reagent into formazan crystals of the cells on the API-loaded scaffolds with respect to those on scaffolds without API.

### Antimicrobial activity

Antimicrobial activity of unloaded and A/S mixture-loaded PHB scaffolds was tested against Gram-positive *S. epidermidis* LMG 10474 and *S. aureus* LMG 8224 analogous to the Kirby-Bauer disc diffusion method [24]. Bacteria were cultured on NA plates (24 h at 37°C) and then transferred to Muller-Hinton (MH) agar plates before performing the test. Sterilized punched fiber mats, 8 mm in diameter were placed on the surface of inoculated MH plates and incubated at 37°C for 24 h. The inhibition zones of *S. epidermidis* LMG 10474 and *S. aureus* LMG 8224 were photographed and the diameter of each growth inhibition ring was measured using ImageJ.

### Statistical analysis

Results of the cell viability assay are shown as mean value ± SD of three independent experiments. Statistical significance in the differences of drug loading and percentage of viable cells, was evaluated by using Student’s *t*-test or one-way ANOVA (Tukey and Scheffe tests) for the single or multiple comparisons of experimental groups, respectively. Difference with *P* values < 0.05 was considered statistically significant. All statistical analyses were performed using SPSS 25.0.

**Figure 2.**
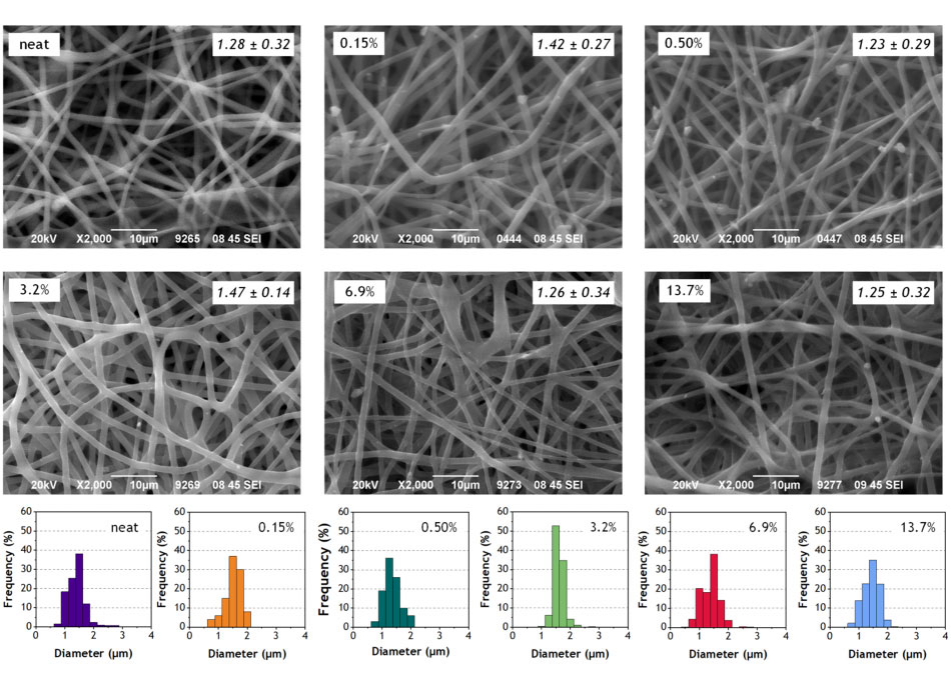
Selected scanning electron micrographs of the neat and A/S mixture-loaded electrospun PHB fibers and their respective diameter size distribution histograms. Percentages refer to the theoretical wt.% of the A/S mixture (see Table 1), while average diameters and SD in µm are also included (see also Table 2).

## Results and discussion

The mixture of A/S pigments has well-established strong wound healing, regenerative and antimicrobial properties, as proven by the authorization by the National Organization for Medicines in Greece and the marketing of the wound healing pharmaceutical ointments, based on the specific APIs, under the trademarks HELIXDERM^®^ and Histoplastin Red^®^. The incorporation of A/S mixture into novel electrospun polymeric fibers as a DDS for topical use, could promote its biological properties. In this concept, we studied the fabrication of electrospun mats, loaded with A/S mixture, using PHB (a non-toxic, biocompatible and biodegradable polymer) and their potential as wound dressings. Different drug loadings were used and the prepared scaffolds were assessed for their physicochemical characteristics, biocompatibility and antimicrobial activity.

### Morphology characterization of the electrospun mats

Electrospinning has gained great attention for the delivery of active molecules, as it produces customizable fibrous mats with release profiles mainly influenced by the structure of the formulated fibers. Taking into account that the morphology of the mats is affected by various parameters during the electrospinning process, such as electric field, feed rate and distance between needle and collector, fibers should be carefully designed with respect to their diameter in order to succeed in obtaining the desired dissolution behavior. Furthermore, process factors, such as the molecular weight of the polymer, electric conductivity, the viscosity of the solution and polymer concentration are also crucial during the preparation of the electrospun fibers [25–27]. In addition, the solvent system used to dissolve the polymer is also important for the procedure [28,29].

Figure 2 shows selected SEM images of all electrospun mats (both neat and drug-loaded), as well as their diameter size distribution histograms. All samples are characterized by fibers with round-shaped cross-sections, with minor drug aggregates on their surface, which suggests that most of the API was homogenously dispersed in the electrospun fibers and a small portion was left upon their surface. As shown in Table 2, the neat polymer sample showed average diameter around 1.28 ± 0.22 μm, while the average fiber diameter of the A/S mixture-loaded samples ranged from 1.25 ± 0.25 to 1.47 ± 0.14 μm. Similar studies report that the addition of drugs into the polymer/solvent solution may significantly affect its properties, mainly the viscosity and the conductivity [30]. As a result, slightly thicker fibers are produced compared to the neat samples [29, 31]. In our case, the introduction of the active ingredients did not substantially change the average diameter of the fibers.

**Figure 3.**
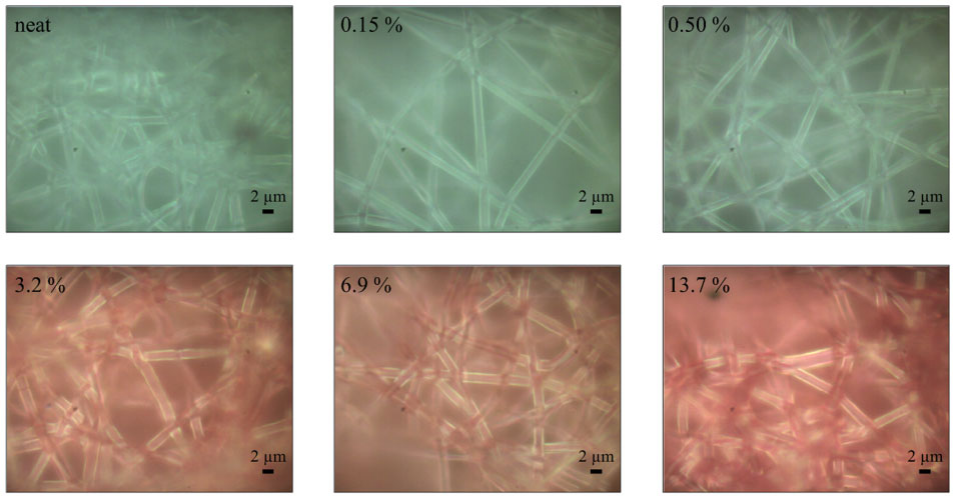
Typical photomicrographs of the A/S mixture-loaded electrospun PHB fibers. Percentages refer to the theoretical wt.% of the A/S pigments mixture (see Table 1).

**Table 2.** Physicochemical characteristics of the prepared fiber mats.

Sample	Mean diameter (μm)	Porosity factor (%)^a^	Water uptake ratio (%)^a^	Entrapment efficiency (%)^a^	Drug loading (%)^a^
Neat	1.28 ± 0.32	76.1 ± 2.6	89.6 ± 3.0	—	—
Sample 1	1.42 ± 0.27	73.0 ± 2.8	78.0 ± 2.4	72.0 ± 1.8	0.11 ± 0.08
Sample 2	1.23 ± 0.29	71.7 ± 2.9	45.6 ± 2.0	85.7 ± 1.2	0.43 ± 0.10
Sample 3	1.47 ± 0.14	69.2 ± 3.0	25.3 ± 1.3	92.6 ± 0.4	2.87 ± 0.13
Sample 4	1.26 ± 0.34	67. 9 ± 3.4	16.5 ± 0.7	52.6 ± 1.0	3.38 ± 0.65
Sample 5	1.25 ± 0.32	65.6 ± 3.6	14.5 ± 0.3	59.2 ± 5.5	7.15 ± 0.60

^a^ Data are shown as mean ± SD of three independent fiber mat samples (in triplicate, *n* = 9 for each sample).

Porosity is an important parameter for tissue engineering scaffolds as it affects cell attachment and proliferation. In general, pores should be sufficiently large to allow gas and nutrient exchange for wound healing and maintain cell homeostasis [32, 33]. Chong *et al*. *[33]* concluded that the preferred porosity for tissue engineering applications should range between 60 and 90%. The porosity factors of the electrospun A/S mixture-loaded PHB scaffolds fabricated in this study are shown in Table 2, where a decreasing trend can be seen, following the increment of the initial amount of A/S mixture used for the preparation of the samples.

This could be attributed to the increasing amount of drug molecules absorbed or loosely bound to the polymer fibers, reducing the overall porosity of the electrospun mats. Furthermore, it should be noted that fabricating well-defined pore sizes through the electrospinning technique is not an easy task, due to the randomly deposited fibers. However, the overall network architecture best mimics the natural ECM, providing an advantage in tissue engineering applications [33].

As depicted in Table 2, the water uptake ratio of the prepared PHB electrospun mats is decreasing as the amount of A/S mixture used for the preparation of the samples and the drug loading increases. Although the porosity reduction would be expected to account for this observation, the porosity is changing from ∼76 to ∼66%, passing from the neat to the most heavily drug-loaded fibers, whereas the water uptake changes from ∼90% to ∼15%, respectively. Therefore, this pronounced diminution in the water uptake ratio may be further related to the high hydrophobic nature of the A/S mixture. Samadian *et al.* [21] reported electrospun cellulose acetate/gelatin nanofibrous wound dressing with even lower water uptake, as suitable to absorb the wound exudates and subsequently improve the wound healing process. These results indicate that the prepared electrospun mats are suitable for tissue engineering applications as wound dressings.

### Entrapment efficiency and drug loading

The incorporation of the drug in each of the prepared samples was estimated and depicted in Table 2. Entrapment efficiencies varied from 52.6% (sample 4) to 92.6% (sample 3). Experimental drug loading on the other hand, ranged between 0.11 and 7.15% (Table 2), with an increasing trend following the increment of the initial amount of A/S mixture used for the preparation of the samples. A/S mixture appeared to have good compatibility with the matrix/solvent system, due to their lipophilicity and high solubility in the solvents used in the experiments and, thus, it could be assumed that the fabricated mats are loaded with most of the initially applied drug. As previously reported [34], when a lipophilic drug (i.e. paclitaxel) highly soluble in the polymer solution (PLLA/chloroform/acetone) was used in electrospinning, the solution jet was rapidly elongated and the solvent evaporated quickly. Thus, phase separation (between the drug and the polymer) was difficult to take place and the drug tended to remain inside the fiber, where sufficient solvent was left. Therefore, when the fiber became dry, the drug was encapsulated inside.

Results in Table 2 depict that the quantity of the A/S mixture incorporated into the PHB electrospun fibers depends on the initial drug used during the preparation of the polymer solutions. All differentiations in the drug loading values were statistically significant, except the ones between samples 1 and 2, as well as samples 3 and 4. As shown, the sample presenting the maximum encapsulation efficiency, was the one containing 3.2% of A/S mixture (theoretical), while further increasing of the API amount results in a significant decrease of encapsulation efficiency. It is important to mention that the entrapment of the A/S mixture in PHB resulted in uniform fiber structures, displaying no significant differences in entrapment efficiencies among different regions alongside their constructs, which is a crucial characteristic. Similar results were observed in our previous work using different polymers [19].

### Optical and spectroscopic analysis

**Figure 4.**
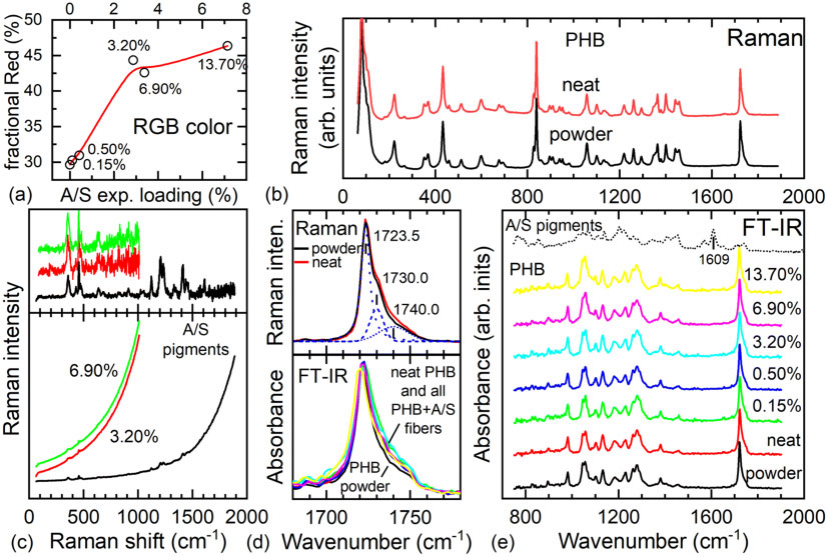
(a) Average fractional red color content of the as recorded micrographs in Fig. 3 as a function of the experimentally determined drug loading (Table 2). the line through data serves as a guide to the eye. (b) Raman spectra of the PHB initial powder material and the electrospun PHB fibers (neat, 0% A/S mixture content). (c) Raman spectra of solid A/S mixture of pigments and two fibers from samples containing A/S pigments mixture (bottom panel), and after subtraction of the luminescent background (top panel). (d) Raman spectra of the powder and neat electrospun fibers (top panel) and IR absorbance spectra of all samples (bottom panel) in the spectral region of the C = O stretching vibration (∼1725  cm^−1^). (e) IR absorbance spectra of all samples in the spectral range 750–1900  cm^−1^. In each plot, all Raman and/or IR spectra are intensity normalized. Labels with percentages inside graphs refer to the theoretical wt.% of the A/S mixture of pigments (Table 1). All Raman spectra were excited using a laser at 515 nm.

Typical photomicrographs of the electrospun samples (neat and drug-loaded mats), acquired from the optical microscope of the Raman instrument, are illustrated in Fig. 3. In agreement with the SEM analysis, fibers in the images appear to have circular cross-sections with similar diameters and minor drug aggregates on their surface. However, the most interesting feature of the optical images is their color. In Fig. 4a, the average fractional red content {*r*/(*r* + *g* + *b*), *r*, *g*, *b* the values for the red, green and blue content of each pixel, respectively} of the as-recorded photographs scales well with the experimentally deduced A/S pigments content (Table 2). It is only the sample of the highest content (13.7% theoretical) that does not comply with the linear trend. Although this may reflect a non-linear response for high pigments content, we have to take into account that neither the illumination (fiber delivered light from a halogen lamp), nor the Raman instrument camera are optimized for color reproduction. However, it is apparent that in the case of bulk production for commercial purposes, optical photography may provide the means for an inexpensive, fast, non-destructive and easily implemented quality control method to assess the A/S pigments' content of the fiber mats.

**Figure 5.**
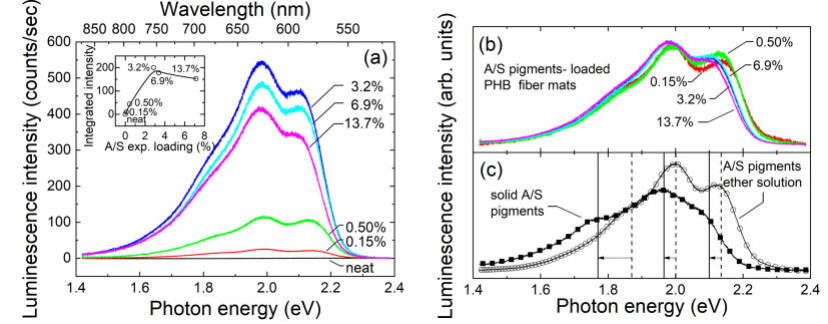
(a) Luminescence spectra of the various electrospun PHB fiber mats measured under the same experimental conditions. The inset depicts their integrated intensities with respect to their experimentally determined drug loading (Table 2). The line through data serves as a guide to the eye. (b) The spectra of (a) normalized to their total intensity. (c) Luminescence spectra of A/S mixture of pigments in diethylether and solid A/S mixture of pigments. Vertical lines and arrows approximately mark the main spectral components and their shifts passing from the solution to the solid sample. Labels with percentages inside figures refer to the theoretical wt.% of the drug (Table 1). All spectra were excited using a laser at 515 nm.

Typical Raman spectra for the initial PHB powder material and the electrospun fibers without any A/S pigments (neat) in the range of ∼100–1900 cm^−1^ are presented in Fig. 4b. Spectra are almost identical to each other and very similar -even with respect to the relative intensities of their peaks- to those found in the literature [35]. However, when the A/S pigments are incorporated in the fibers, a strong luminescent background appears (Fig. 4c, bottom panel) that does not allow the acquisition of high-quality Raman spectra (Fig. 4c, top panel). The luminescent background originates from the solid A/S pigments mixture, as verified by their corresponding spectrum. In the case of the fiber samples, it is further blue-shifted, making the acquisition of Raman signal even more difficult. Raman spectra were excited using extremely low laser power values to avoid photoinduced alterations of the drug molecules. Consequently, the PHB spectral signature is not evident in the acquired spectra, unlike the stronger signal of the A/S pigments, due to their resonant Raman excitation. Indeed, the spectral profile of the API-loaded fibers, including the luminescent background, with more intense Raman peaks at approximately 360, 457, 1122, 1206, 1233, 1410, 1609 cm^−1^, strongly resembles that of alkannin [36–38], although peaks are clearly broader and in some cases they merge. It was possible to detect the presence of a noisy A/S peak at ∼360 cm^−1^, even for the sample with 0.5% A/S pigments (not shown). Therefore, successful drug incorporation in the PHB fibers is verified by Raman spectroscopy but the excessive noise to signal values due to the background do not allow comparison of the various Raman spectra and possible detection of any molecular conformation differences or reliable quantitative information from the spectra concerning the A/S pigments. On the other hand, careful inspection of the Raman spectra of the PHB powder and the neat electrospun fiber mat in the spectral region around 1725 cm^−1^ reveal small differences in their respective spectral profiles (Fig. 4d, top panel). This spectral region, attributed to the C = O stretching vibration, has been used for the characterization of the crystalline state of PHB-based materials by means of both Raman [35] and FT-IR [39] spectroscopies. In both cases, the fitting of spectral profiles is possible by the use of three peak line shapes, in our case at ∼1724, 1730 and 1740 cm^−1^, the first two assigned to the crystalline phase of PHB and the last one to its amorphous one (Fig. 4d). It is clear that the crystal phase of PHB is the dominant phase and that the electrospun PHB (neat) is slightly more amorphous than the initial PHB powder material. This observation is further confirmed by IR spectroscopy.

The intensity normalized FT-IR spectra for all the samples (Fig. 4e), in a similar spectral range with that of the Raman ones, are dominated by the IR absorption of PHB and are spectrally the same with each other and those in the literature [40], irrespectively of their A/S content. Unlike the case of Raman spectroscopy, in the IR spectra, the spectral region around the C = O stretching band of PHB (∼1725 cm^−1^) can be recorded for all samples, including the drug-loaded fibers (Fig. 4d, bottom panel). The relative intensity of the peak related to the amorphous phase with respect to the peaks of the crystalline phase in all the fiber mats, is slightly, but consistently, higher than that in the case of the initial powder material. Therefore, it appears that the electrospinning process of PHB results in a small increase of its amorphous content for all A/S-loaded scaffolds and thus, the observed differences in their API release cannot be attributed to their degree of crystallinity. No systematic changes were observed in the crystallinity of PHB with respect to the A/S pigments content. Finally, the A/S pigments were almost undetectable in the IR spectra. The 1609 cm^−1^ peak of the A/S pigments, appearing in a featureless spectral region of the PHB spectrum, may serve as a telltale feature for the presence of the drug. However, the absorption due to the A/S pigments in the concentrations under investigation appears to be negligible and a spectral feature close to the noise level at this frequency was detected only for the case of the maximum concentration. Therefore, IR spectroscopy is not suitable for detection and quantification of the A/S pigments in our case.

As mentioned above, the acquisition of Raman spectra was hindered by a luminescent background, appearing more disturbing in the case of the loaded fiber mats than that of solid A/S pigments. In order to clarify the observation, the luminescence spectra of the fiber mat samples were recorded and are presented in Fig. 5a. Evidently, the neat sample does not exhibit any luminescence, contrary to the A/S containing fibers, where three main bands constitute the experimental spectra, at approximately 1.98, 2.12 and 1.85 eV, in decreasing apparent intensity order. The integrated intensities of the luminescence spectra (Fig. 5a, inset) appear to depend quasi-linearly on the experimentally deduced A/S pigment content up to a value of ∼3%, where the signal seems to saturate, or even decrease. Although, for low drug contents, increasing concentration should in principle increase the signal, at considerably higher concentrations the probed volume may be reduced because the excitation radiation cannot penetrate as deep as before, leading to a saturation effect. However, this may not be the only cause. Normalizing the spectra-dividing by their integrated intensity (Fig. 5b)-makes clear that the samples of higher concentration are red-shifted with respect to those from more dilute samples. The spectrum of the solid A/S pigments is further red-shifted (Fig. 5c). The most characteristic behavior is exhibited by the spectral component at the highest photon energy that is clearly visible at lower concentrations, loses intensity relative to the central peak and red shifts in samples of higher concentrations and eventually represents a bump in the spectral profile of the solid A/S pigments. To clarify the differences, the luminescence of an A/S pigments diethylether solution has also been measured and presented in Fig. 5c. It is apparent that the spectrum of the drug dispersed at the molecular level (i.e. the solution) resembles better the spectra of fiber mats of low drug concentrations, whereas those from samples of higher concentration appear closer to the spectral profile of the solid mixture. Therefore, we attribute the observed differences to a dielectric confinement effect, without excluding other possible interactions of the molecules with their environment (e.g. hydrogen bonding). High drug concentration near the fiber surface or a surface layer of attached drug molecules will change the effective environment of the molecules shifting their luminescence’s spectral features and altering their relative intensities. This may affect the intensity dependence on the drug content that was observed to change for high concentrations. Further, a higher number of drug molecules bound at or near the fiber surface is also compatible with the initial burst observed in the release kinetics of the samples (*vide infra*).

**Figure 6.**
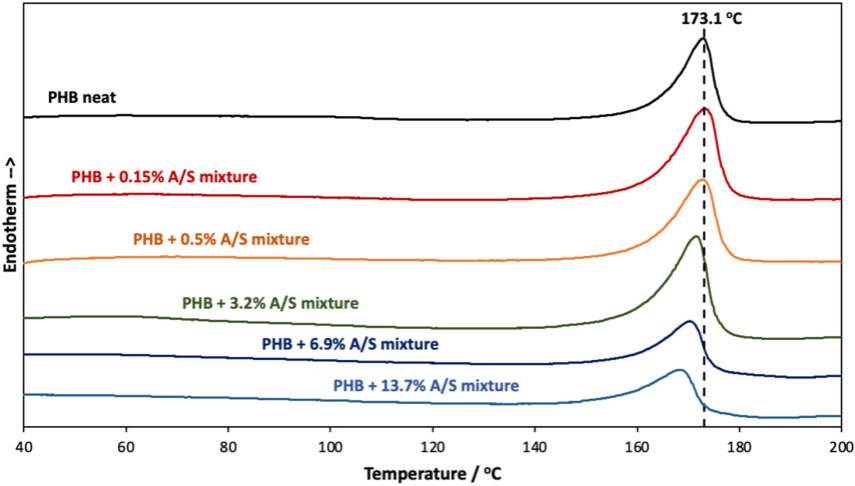
DSC thermographs for the neat PHB and the composite fibrous membranes.

### Thermal analysis

As shown in Fig. 6, differential scanning calorimeter runs revealed that the neat polymer fibrous membranes present an endothermic peak, identified as melting point, at 173.1°C. The addition of A/S mixture results in a melting point depression, which becomes significant at API’s content higher than 3.2 wt.%. Such small melting point depression is a typical behavior of the encapsulation of small molecules inside the polymer matrix, which hinders the development of large pure polymer crystallites and facilitates melting upon the addition of thermal energy.

**Figure 7.**
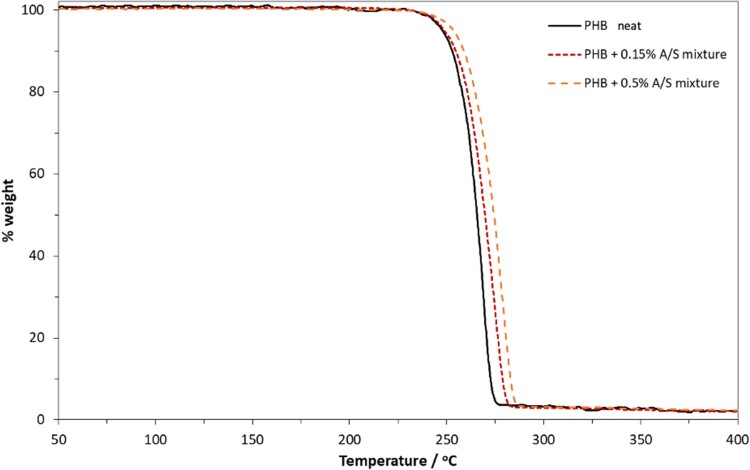
TGA thermographs for the neat PHB and the composite fibrous membranes with 0.15 and 0.5 wt.% A/S mixture.

As shown in Fig. 7, which presents the TGA thermographs for the neat polymer and the membranes loaded with 0.15 and 0.5 wt.% A/S mixture (theoretical values), the pure PHB membranes present constant weight up to 225°C, while the addition of the APIs, even in small concentrations, results in the enhancement of thermal stability, suggesting their successful encapsulation.

**Figure 8.**
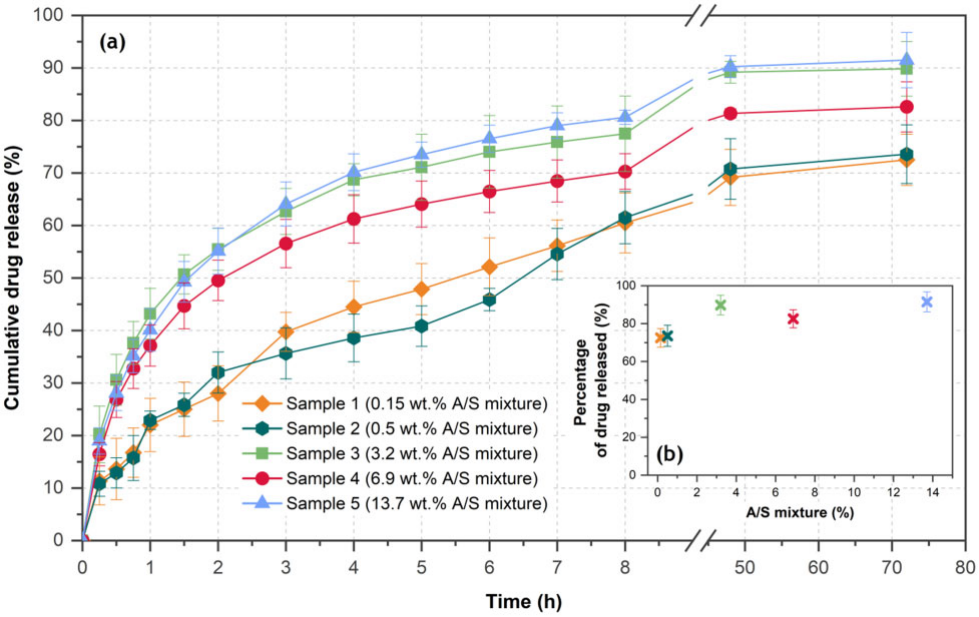
(a) Drug release profile of the encapsulated drug with time and (b) percentage of drug totally released from PHB electrospun fibers vs nominal drug loading. Each value represents the mean ± SD of three independent experiments in triplicate (n = 9 for each sample).

### Drug release studies

Figure 8a shows the time evolution of the cumulative amount of the A/S mixture released from the drug-loaded PHB fibers, divided by the total encapsulated drug quantity. The fraction of the drug finally released showed variations from sample to sample (as depicted in Fig. 8b), related to the total drug content of the fibers. Specifically, the samples with the lower drug content (samples 1 and 2 with drug loadings estimated to 0.11 and 0.43 wt.%, respectively) released 72.5% and 73.6% of their total drug within 72 h, whereas, during the same period, the sample with the highest drug content (sample 5, 13.7 wt.%) released 91.5% of its entrapped drug.

**Figure 9.**
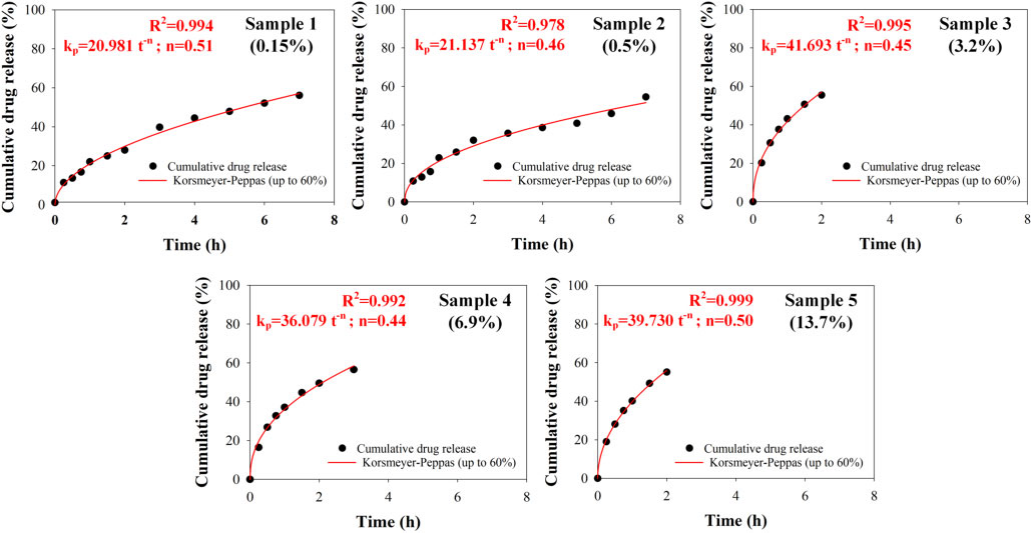
Fitting of drug release profile of all drug-loaded PHB samples with the Korsmeyer–Peppas model, for up to 60% of totally released drug.

Carefully inspecting Fig. 8a, for the samples of higher nominal drug loading (3.2, 6.9 and 13.7 wt.%), a fast initial release rate of the A/S mixture is observed for the first 2 h, when more than 50% of the drug has been released. This finding may be explained by assuming that a part of the drug molecules are absorbed or loosely bound on, or near, the surface of the polymer fibers, thus they could be easily diffused into the release medium leading to this burst effect. Analogous release profiles for similar APIs were reported from PCL/PTMC fiber mats [41]. For the fiber mats with lower drug loading (0.15 and 0.5 wt.%), a smoother release rate is observed, requiring approximately 8 h for the release of more than the 50% of the encapsulated drug. In all samples, the initial faster release was followed by a slow release rate, reaching a plateau after 48 h. Similar findings have been previously reported for fiber meshes loaded with several drugs [19, 42–44].

Polymer matrices tend to release encapsulated drugs via mechanisms such as drug diffusion, matrix erosion, swelling, or a combination of these, and the dominating mechanism is determined by numerous factors (polymer composition, *M*_w_ and crystallinity, drug physicochemical properties, matrix–drug interaction and morphology, porosity etc.). In the case of biodegradable matrices, the phenomenon is even more complex, due to alterations in polymer phase properties during degradation, leading to changes in drug diffusivity and permeability with time [43].

In order to gain an insight into the mechanism of the A/S mixture’s release from the prepared electrospun mats, the obtained dissolution data were fitted with the power law model of Korsmeyer–Peppas [45]. According to this model, the time evolution of the released drug can be described by *D_t_*/*D_∞_* = *k_p_*·*t^n^*, where *D_t_* is the amount of drug released up to time *t*, *D_∞_* is the amount of drug released as time approaches infinity, *D_t_/D_∞_* is the fraction of drug released at time *t*, *k_p_* is a kinetic parameter, characteristic of the drug-polymer system, and *n* is the diffusional exponent parameter that characterizes the release mechanism. This is a versatile model because for *n *=* *0.5, it actually becomes the extensively used Higuchi model, describing the Fickian release mechanism of drugs from an insoluble matrix when the loading of solute exceeds its solubility in the surrounding fluid [46], whereas for *n *=* *1 it becomes the zero-order model (linear, without an offset), where the drug release rate is constant. Values of *n* higher than 0.5 describe non-Fickian release mechanisms. However, this value applies for thin film geometries of the releasing device, whereas for cylindrical or spherical geometries the values become 0.45 and 0.43, respectively [47, 48]. It must be noted that this model and parameter interpretations apply as long as data for up to 60% released drug fractions are used, a prescription not taken into account in many studies.

In Fig. 9, the experimental release data up to the 60% of released drug were fitted using the Korsmeyer–Peppas model. The *n* parameter varies, non-systematically, between 0.44 and 0.51. It must be noted that our system in the micrometric scale has a cylindrical symmetry (fiber), whereas macroscopically, a plane symmetry is attained (densely entangled fibers to form the mats). For the former, an *n* value of 0.45 is expected, whereas 0.50 is the predicted values for the latter. Consequently, our fitting results indicate that the dominant A/S mixture’s release mechanism for all PHB electrospun fiber mats is of Fickian nature. Furthermore, the initial burst observed for the highly drug-loaded fiber mats, attributed to a part of the drug molecules being absorbed or loosely bound on, or near, the surface of the polymer fibers, is reflected in the *k_p_* parameter values that are almost doubled passing from the low A/S mixture sample concentrations to the higher ones.

**Figure 10.**
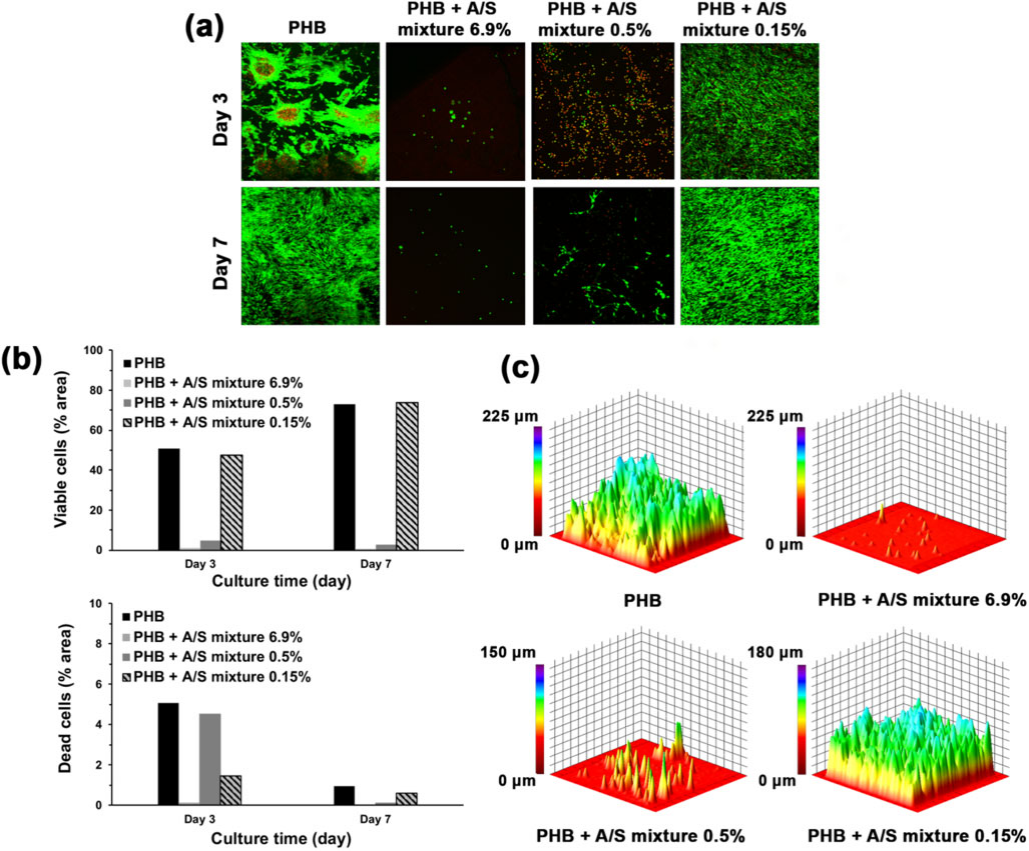
Confocal microscopy images: (a) live/dead staining of Hs27 cells seeded on A/S mixture-loaded PHB electrospun scaffolds. Live/dead assay stains live cells green, dead cells red (nuclei only). (b) Analysis of surface area covered by viable (up) and dead (down) cells at days 3 and 7. (c) Representative 3D-constructed confocal microscopy images of alive Hs27 fibroblasts on scaffolds after 7 days of culture. Confocal images were recorded at 10× magnification.

Overall, the observed release profile, despite its initial quick pace for samples with higher API concentration, could be considered as an ideal drug-release profile for numerous medical applications (such as wound healing), when an immediate therapeutic effect is required followed by a prolonged action. For example, in the treatment of ‘fresh’ or infected wounds and injuries, where more drug is required rapidly to prevent and actively treat wound infections, dressings with higher API loading are preferable, since they exhibit an enhanced burst effect. Consequently, after 1–2 days, the same wound could be treated with a different dressing of lower A/S content, lower burst release and a much smoother, gradual and prolonged release rate, retained for several days. This would enhance epithelization and tissue proliferation, without the need of replacing it, ideally biodegrading in place.

In relation to the binding-linkage type of the A/S mixture with the PHB, it can be hypothesized that based on the functional groups of the PHB and A/S mixture, the PHB carbonyl groups and the −OH groups of the A/S mixture form hydrogen bonds, as proposed for PHB and keratin containing amide groups [49].

### Biocompatibility evaluation

#### Cell adhesion

Τo assess the biocompatibility of PHB electrospun scaffolds in the presence of the bioactive molecules, A/S and their esters, we decided to examine certain characteristics that are crucial in the wound healing process, such as cell attachment, surface coverage and infiltration of human skin fibroblast Hs27 cells [50]. Fig. 10a and b shows that the neat and 0.15% A/S mixture-loaded PHB scaffolds achieved greater living-cell coverage compared to the 0.5 and 6.9% samples on both days 3 and 7, with the 0.15% sample achieving an even more uniform cell distribution and satisfactory cell penetration after 7 days of culture compared with the neat fiber mat (Fig. 10c). Specifically, the 0.15% A/S mixture-loaded scaffold at day 7 (Fig. 10b) reached approximately 70% of living-cell surface coverage, slightly surpassing PHB neat scaffold and thus, suggesting that A/S at low concentrations might have a positive effect on cell spreading. It is noteworthy that in the case of the 6.9% A/S mixture-loaded mat on day 3, it showed the lowest cell attachment and growth among all samples (1.2% coverage of the scaffold area), that were further reduced on day 7 (0.6% coverage of the scaffold area).

**Figure 11.**
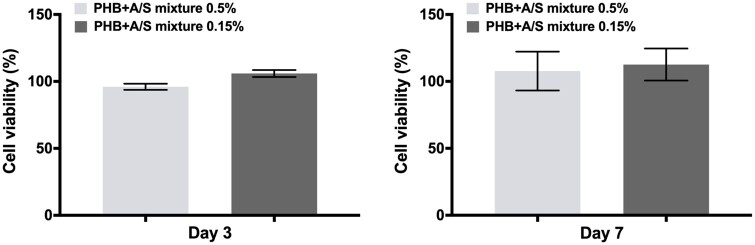
Cell viability evaluation of Hs27 cells on the A/S mixture-loaded PHB scaffolds by MTT assay after 3 and 7 days. Percentages (%) represent normalized values of each scaffold based on the neat sample (control) for each time point. Data are shown as the mean of three individual experiments ± SD.

In addition, the conspicuous number of dead cells present in the 0.5% A/S mixture-loaded PHB scaffold, during the first 3 days, had been diminished by day 7, indicating the non-toxic nature of the drug-loaded scaffold. Regarding neat PHB meshes, a minor population of dead cells was observed, accounting for 5% of the scaffold’s area on day 3 (Fig. 10b down), that could be attributed to the environment of the scaffold. This might have caused some type of shock to the cells, which nevertheless adapted to the mats’ presence on day 7 (increased coverage by alive cells and decreased by dead cells). It is evident that the addition of A/S mixture in the PHB fiber mats led to an inversely concentration-dependent cell adhesion, with the lowest drug concentration demonstrating a beneficial effect, whereas higher concentrations had a negative impact on cell attachment, proliferation and uniform distribution. A tentative explanation to this might be the fact that PHB is by nature a hydrophobic material [51], thus by increasing the concentration of lipophilic A/S derivatives in the scaffolds, the fibrous surface becomes more and more hydrophobic, which in turn hinders cell adhesion and proliferation [52].

#### Cell morphology

Based on the results from cell adhesion evaluation, we decided to proceed with neat, 0.15% and 0.5% A/S mixture-loaded PHB scaffolds (theoretical values), in order to assess the morphology of the attached cells. SEM images confirmed our confocal microscopy data showing good attachment and spreading of Hs27 fibroblasts, specifically on the neat and 0.15% A/S mixture-loaded scaffolds (not shown). Already from the third day of culture, the fibrous matrices favored the formation of a dense cellular network along with the deposition of ECM (seen as surface coating). The attached cells retained their fibroblast-like morphology displaying elongated and bipolar/multipolar shapes.

#### Cell viability

To confirm the biocompatibility of the attached Hs27 on the surface of the fabricated scaffolds (unloaded and loaded), we examined the cell viability by MTT assay after 3 and 7 days of incubation. Figure 11 shows that the viability of Hs27 fibroblasts on PHB scaffolds was maintained at a high level throughout the seven days of culture. The MTT assay results are in agreement with the confocal microscopy analysis (Fig. 10) by showing a consecutive cell growth pattern between days 3 and 7, albeit the differences between the samples are less pronounced. In more detail, drug-loaded scaffolds showed good cytocompatibility in the respective used concentrations, with the 0.15% fiber mat exhibiting higher cell viability at day 3 compared to the 0.5% loaded fiber mat, while after 7 days both A/S mixture-loaded PHB fiber mats enhanced cell viability. Such results are in support of a previous study, where poly(3-hydroxybutyric acid-co-3-hydroxy valeric acid) (PHBV) nanofibers (10% w/v polymer), loaded with curcumin (0.1%–0.5% w/v) as active compound, demonstrated excellent biocompatibility with L929 fibroblast cells [53]. Similarly, two other groups tested the effect of polyvinyl alcohol (PVA) [54] and gelatin/PVA/chitosan [55, 56] electrospun nanofibers containing *Lithospermum erythrorhizon* extract alone or in combination with curcumin, on L929 fibroblasts during an *in vitro* indirect cytotoxicity assay. Their results showed that the scaffolds acted beneficially on cell viability and in some cases, enhanced cell proliferation was observed.

### Antimicrobial activity

A/S and their derivatives have been long established as antimicrobial agents against a plethora of bacterial strains [17]. *S. epidermidis* and *S. aureus* are both *Gram*-positive bacteria that normally occur on the skin, as part of the human microbiota. Nevertheless, it has been reported that in cases of implanted medical devices, *S. epidermidis* may facilitate infections by entering the surgical site and rapidly accumulating, which leads to the formation of biofilms and thus, to significant complications [57]. Additionally, *S. aureus* has become one of the most common infections related to skin wounds and, if left untreated, may lead to serious complications [58]. To this end, the antibacterial activity of neat and A/S mixture-loaded PHB electrospun scaffolds was evaluated by the disc diffusion method (Table 3). After 24 h of incubation of *S. epidermidis* LMG 10474, inhibition zones were observed around the edges of the 3.2, 6.9 and 13.7% A/S mixture-loaded fiber mats, with the sample with 13.7% theoretical content displaying the greatest inhibition. Similarly, in the case of *S. aureus* LMG 8224, the 3.2, 6.9 and 13.7% A/S-loaded scaffolds yielded comparable inhibition halos. On the other hand, fiber mats with 0.15% and 0.5% A/S mixture did not show any measurable inhibition for any of the two bacteria, probably due to the lower concentration of the drug. Absence of inhibition zone was observed for the neat PHB scaffold, as well. The mixture of A/S used as API for fibers’ preparation showed a comparable antibacterial activity to the drug-loaded fiber membranes for *S. epidermidis* LMG 10474 and *S. aureus* LMG 8224 (appx. 10.2 mm inhibition zone for 30 μg API and 13.7 mm for 300 μg, for both bacteria). Han et al. have also demonstrated the antibacterial activity of PCL/PTMC electrospun nanofibers loaded with shikonin (1 wt.% and 5 wt.% based on the weight of PCL/PTMC) against the *Gram*-positive pathogen, *S. aureus* and the *Gram*-negative, *Escherichia coli* [41].

**Table 3.** Antimicrobial activity of neat PHB fibers, A/S mixture-loaded PHB fibers and the mixture of A/S against *S. epidermidis* LMG 10474 and *S. aureus* LMG 8224.

Zone of inhibition (mm)							
Bacterial strains	A/S mixture (30 μg)	PHB neat	PHB + A/S mixture 0.15%	PHB + A/S mixture 0.5%	PHB+A/S mixture 3.2%	PHB+A/S mixture 6.9%	PHB+A/S mixture 13.7%
*S. epidermidis* LMG 10474	10.29 ± 0.20	—	—	—	11.55 ± 0.37	10.02 ± 0.18	11.81 ± 0.42
*S. aureus* LMG 8224	10.22 ± 0.75	—	—	—	10.84 ± 0.36	9.62 ± 0.76	13.72 ± 1.15

(–) indicates that no zone of inhibition was observed.

## Conclusions

To our knowledge, this is the first study reporting the use of PHB as a biomaterial for loading the bioactive compounds alkannins and shikonins by means of electrospinning. The obtained fiber mats exhibited a defect-free morphology with the average fiber sample diameters ranging between 1.25 and 1.47 μm. Optical spectroscopies suggest that the PHB powder is mainly crystalline and only marginal increase of the amorphous phase takes place in the fibers, due to the electrospinning process. No systematic changes were observed in the crystallinity of PHB fibers with respect to the A/S pigments content. Furthermore, it appears that for the small theoretical concentrations, the drug is efficiently encapsulated in the fibers, whereas a higher drug content bound at or near the fibers’ surface is suggested for the samples of higher nominal concentrations. For all samples, a series of physico-chemical characteristics were assessed indicating a good drug-polymer compatibility, hence suggesting that A/S mixture-loaded PHB fiber mats could potentially serve as a promising drug delivery application within the field of skin tissue regeneration. In addition, certain samples were selected for examining their *in vitro* biological profile by seeding Hs27 fibroblasts on the surface of the scaffolds for an incubation period of 3 and 7 days. The findings revealed that the amount of A/S mixture incorporated in the fibrous matrix positively affected cell adhesion and distribution in an inversely concentration-dependent manner. Notably, the 0.15% scaffold facilitated cell attachment and enhanced cell proliferation. Moreover, for the neat and samples with 0.15–0.5% A/S mixture, no cytotoxicity was observed at the Hs27 fibroblasts, therefore confirming the results from SEM and confocal microscopy measurements. Finally, scaffolds containing 3.2, 6.9 and 13.7 wt.% A/S mixture demonstrated their antibacterial activity against *S*. *epidermidis* and *S. aureus*, which is a critical trait of any wound healing application. The antibacterial action of scaffolds measured is attributed to the A/S mixture encapsulation.

Our data provide additional information about A/S-loaded biomaterials and their use, thereof, as advanced medical devices. It is of great importance to take advantage of the strong wound healing potential of such well-approved and clinically applied biological agents and improve their pharmacokinetic parameters. Future research should further develop and confirm the findings of the present study by taking the next step and proceed to *in vivo* testing. Studies of our group on tissue engineering scaffolds with other polymers and A/S as APIs are in progress, along with structure-activity relationship studies and will be soon reported.
